# *In situ* nuclear matrix preparation in *Drosophila melanogaster* embryos/tissues and its use in studying the components of nuclear architecture

**DOI:** 10.1080/19491034.2022.2043608

**Published:** 2022-03-03

**Authors:** Rashmi U Pathak, Ashish Bihani, Rahul Sureka, Parul Varma, Rakesh K Mishra

**Affiliations:** a Centre for Cellular and Molecular Biology, Uppal Road, Hyderabad, India; b Embl, Rome, Italy; c Present Address: Department of Neuroscience, Development and Regenerative Biology, The University of Texas at San Antonio, Texas, USA; d Tata Institute for Genetics and Society, Bangalore, India

**Keywords:** Nuclear matrix, nuclear architecture, *in situ* NuMat

## Abstract

The study of nuclear matrix (NuMat) over the last 40 years has been limited to either isolated nuclei from tissues or cells grown in culture. Here, we provide a protocol for NuMat preparation in intact *Drosophila melanogaster* embryos and its use in dissecting the components of nuclear architecture. The protocol does not require isolation of nuclei and therefore maintains the three-dimensional milieu of an intact embryo, which is biologically more relevant compared to cells in culture. One of the advantages of this protocol is that only a small number of embryos are required. The protocol has been extended to larval tissues like salivary glands with little modification. Taken together, it becomes possible to carry out such studies in parallel to genetic experiments using mutant/transgenic flies. This protocol, therefore, opens the powerful field of fly genetics to cell biology in the study of nuclear architecture.

**Summary**: Nuclear Matrix is a biochemically defined entity and a basic component of the nuclear architecture. Here we present a protocol to isolate and visualize Nuclear Matrix *in situ* in the *Drosophila melanogaster* and its potential applications.

## Introduction

It is well established that nucleus is compartmentalized, and its functional domains are dynamically linked. Nuclear matrix (NuMat) is a structural framework involved in organization of internal nuclear architecture. NuMat was first described by Berezney and Coffey in 1974 as a nuclear substructure consisting of a meshwork of ribonucleo-proteinaceous filaments, which resists extraction by nonionic detergents and high salt concentrations. Since its inception, many methods to isolate this entity have been developed and refined. However, skeptics continue to question whether NuMat represents an accurate *in vivo* situation. There are arguments that the genome is self-organizing and contains information for its own quarternary structure in interphase nucleus and the chromosomes maintain their territory due to steric hinderance between the chromosome surfaces. This bring forth the pertinent question that the genome being invariable, how does it fold differently in cell-type-specific manner? The observation that a ribo-proteinaceous scaffold persists in the nucleus even after chromatin has been completely removed, seeded the idea of NuMat [1]. This scaffold was proposed to play a central role in dynamic nuclear organization. Much to support the idea, further work from Berezney’s group elucidated that the chromosome territories remain intact even when most of the histones were removed resulting in loss of chromosome structure. The territories persist up to a point till NuMat proteins are released [2]. In parallel, studies from Laemmli’s group showed that a backbone of non-histone proteins is responsible for mitotic chromosome structure and the scaffold organizes the DNA into loops along its length [3]. Unifying the two comparable structures, namely NuMat and MiCS (Mitotic chromosome scaffold), work from our group has shown that they have many proteins in common [4]. These studies and many more altogether have brought the NuMat center-stage where it can be endorsed as a ribo-proteinaceous structure that packages the DNA to set up a cell-type specific 3D organization in the nucleus. It truly is a biochemical snapshot of nuclear organization and persists through mitosis to convey architectural information to daughter cells after cell division. Apart from packaging, several studies have proposed NuMat as a substratum on which various nuclear processes such as replication, transcription, DNA repair, splicing and chromatin remodeling can happen. It is shown that NuMat association of the components involved in these processes indeed facilitates these nuclear processes [5–9].

On the virtue of such compelling evidences, we maintain the idea of NuMat as a nuclear substructure that acts as a dynamic support for all nuclear functions and present this methodology for its *in situ* observation. Till now, most of the studies to elucidate biological relevance of NuMat have been carried out either in cultured cells or isolated nuclei [10–12]. Such studies provide valuable insights but have the caveat of disturbing the nuclear architecture and genome organization and hence may not reflect the *in vivo* conditions faithfully. Our method fills the gap and has the potential to be a powerful means to define and analyze the components of NuMat in biologically relevant setup.

The utility of our method increases manifold as it can be used in conjunction with genetic experiments. Till now, it has been challenging to harness the power of *Drosophila* genetics in the study of NuMat because of the lack of a methodology to visualize NuMat in the context of the intact organism or tissue. Our method permits the preparation of NuMat *in situ* in the developing embryo and larval tissues of *D. melanogaster* and makes it possible to visualize NuMat *in situ* in the organism. *D. melanogaster* is one of the preferred model organism for variety of reasons, including the sheer abundance of available mutants. *Drosophila* genetics combined with the presented method of *in situ* NuMat preparation has the potential to provide a robust method to study various components of nuclear architecture.

## Results

### *In situ* NuMat preparation retains the characteristic features of nuclear architecture

**Figure 1.**
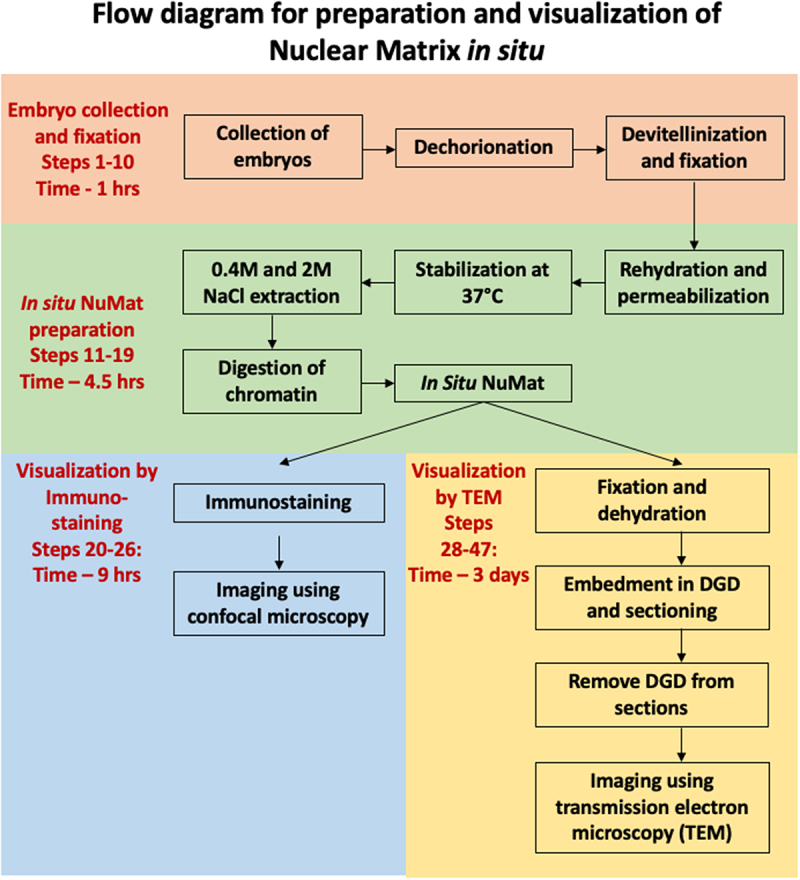
Flow diagram elucidating the main experimental steps of in situ NuMat preparation.

The main experimental steps of *in situ* NuMat preparation have been outlined in the form of a workflow in Figure 1. In an intact nucleus, the NuMat is concealed by dense chromatin mass which is removed by extraction with nonionic detergent and salt followed by DNase I digestion. The NuMat thus revealed, consists of a nuclear lamina, an internal matrix composed of thick polymorphic fibers and ribonucleoprotein particles and remnants of nucleoli. To assess the quality of nuclear matrices prepared by the *in situ* NuMat preparation method, we visualized it by TEM and confocal imaging (Figure 2).

**Figure 2.**
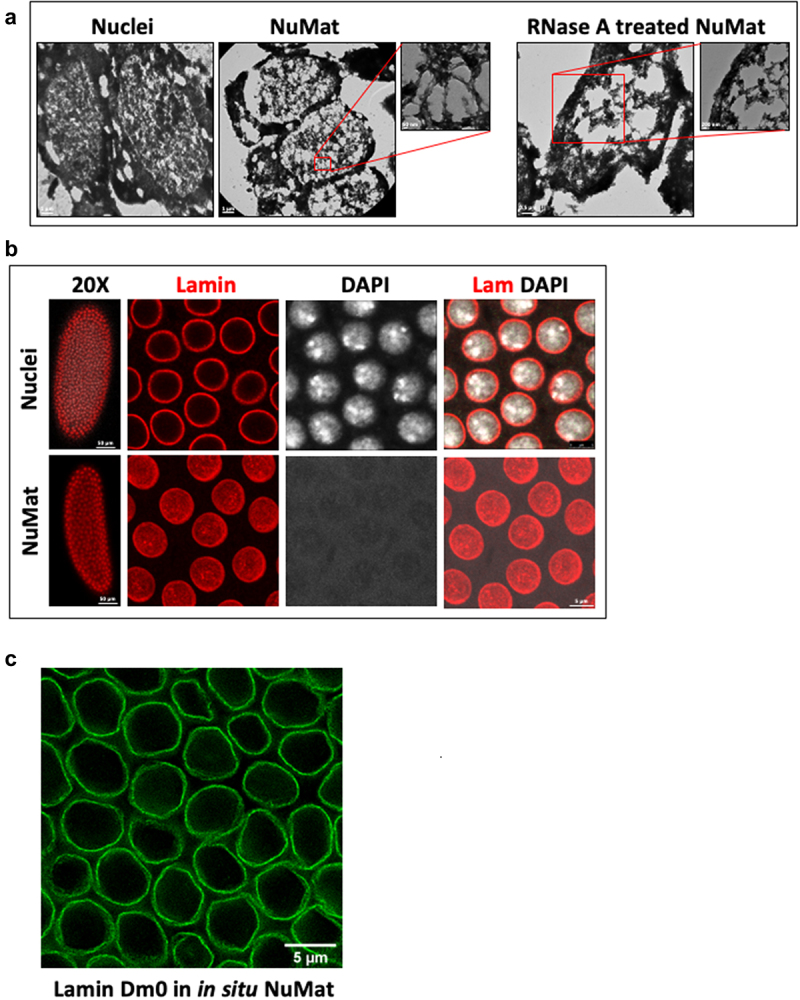
**Visualization of *in situ* NuMat**. *D. melanogaster* embryos at early stages of development (0–2 hr) were used to prepare nuclear matrices *in situ*. **A**. Visualization by TEM. Images obtained by TEM of resinless sections of embryos carrying intact nuclei, *in situ* NuMat and RNase A treated *in situ* NuMat. The fine filaments seen in NuMat, are lost upon RNase A treatment, leaving large gaps in the nuclear structure and leading to collapse of nuclei. **B**. Visualization by confocal microscopy. Unextracted embryo and embryo with *in situ* NuMat, were immuno-stained with anti-Lamin Dm0 and DAPI and imaged by confocal microscopy. In unextracted embryos, Lamin Dm0 appears as a ring at the nuclear periphery of intact nuclei. After *in situ* NuMat preparation, no DAPI staining is observed in the nucleus, as chromatin has been digested and extracted out. Lamin Dm0 staining can now be seen in the nuclear interior as well. **C**. STED visualization of Lamin Dm0 stained *in situ* NuMat. **D**. Remnants of nucleolus remain associated with *in situ* NuMat. Confocal images of unextracted embryo and embryo with *in situ* NuMat, were immuno-stained with anti-Fibrillarin, anti-Lamin Dm0 and DAPI. Loss of DAPI staining indicates extraction of chromatin. *In situ* NuMat shows prominent staining with fibrillarin indicating remnants of nucleolus remain associated with the nuclear substructure. **E**. *In situ* NuMat prepared without crosslinking, without stabilization or with over-crosslinking. *In situ* NuMat is efficiently prepared as evident by absence of DAPI staining, even when the embryos are not crosslinked or are not stabilized. The circular morphology of the nuclei remains intact, but internal lamin staining is not visible. Over-crosslinking results in clumps of DNA that remains unextracted as visualized by DAPI staining. Internal lamin staining is also not sufficiently revealed. All of the confocal image were acquired using a Leica SP8 confocal microscope. The whole embryo images were taken with 20X objective and the higher magnification images were taken with 63X objective. Images were processed using LAS X software from Leica.

**Figure 2.**
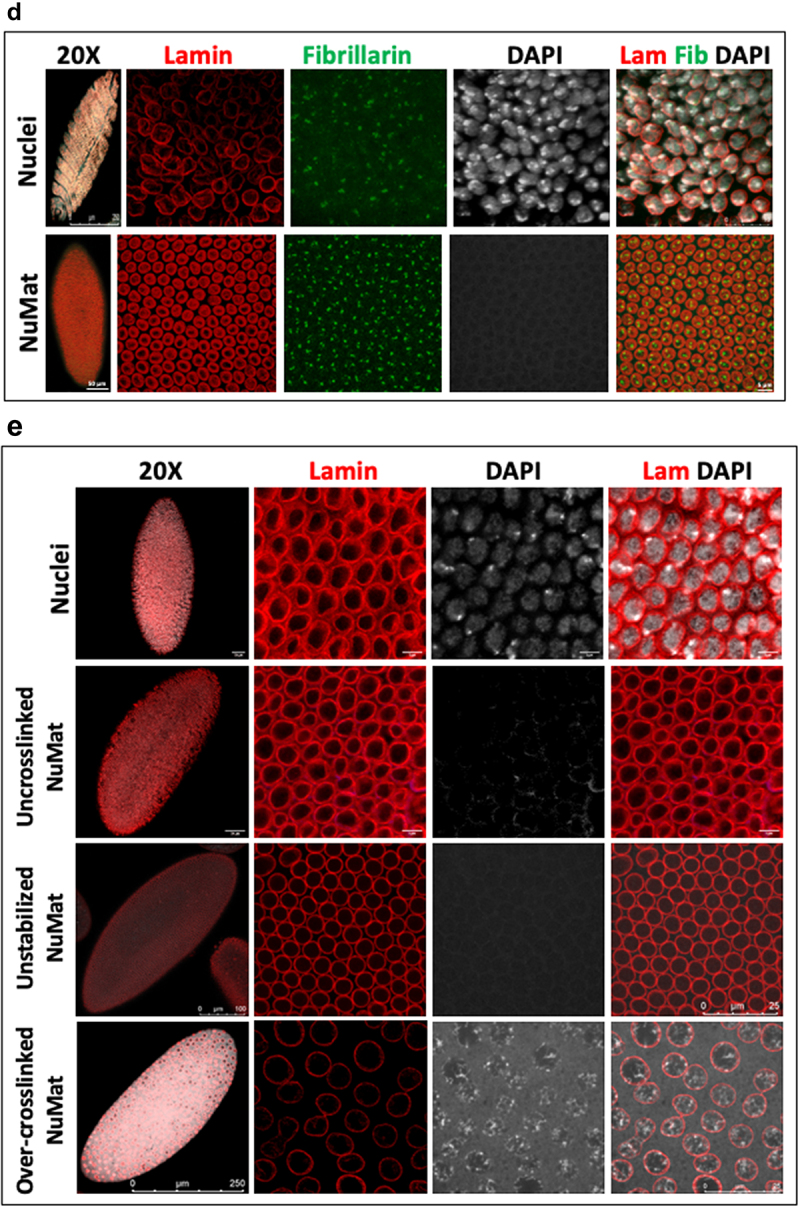
Continued.

Early studies have shown that the filamentous network in an extracted nucleus is best illustrated when embedment free sections are visualized by TEM. The conventional epoxy embedded sections can obscure important biological structures; thus, resinless sections have been routinely used to visualize NuMat. We used resinless sectioning followed by TEM imaging to assess the ultrastructure of *in situ* NuMat prepared in early *Drosophila* embryos (Figure 2a). In line with previous reports, the intact nucleus is filled with a dense network of chromatin and soluble proteins. After extraction with detergent, salt and DNase I, the nuclear interior is visualized as a network of filaments bound by lamina. Treatment of NuMat with RNase A results in loss of the internal fibers and aggregation of ribonucleoprotein particles, highlighting the importance of RNA in matrix organization. The RNA-containing NuMat appears as a self-supporting three-dimensional structure, which collapses after removal of RNA. The RNA depleted NuMat is also markedly distorted in overall shape. These observations suggest that the nuclear matrices prepared *in situ* in intact *Drosophila* embryos are ultra-structurally similar to NuMat prepared in cultured cells by traditional methods.

The chromatin depleted NuMat is a core structure where lamins are retained along with a unique set of nuclear non-histone proteins that resist salt and detergent extraction. To visualize these proteins, we immuno-stained the *in situ* NuMat with anti-Lamin Dm0 and anti-Fibrillarin antibodies (Figure 2b, 2c and 2d). We observe that Lamin Dm0 grossly defines the nuclear morphology. Figure 2c shows a single z-section image of *in situ* NuMat taken by higher resolution STED (stimulated emission depletion) microscopy. The image shows NuMat features at higher spatial density and Lamin Dm0 distribution from nuclear periphery to interior is clearly evident. Apart from forming a meshwork adjacent to the inner nuclear membrane, it is also present in the nuclear interior. Further we also see that the well-characterized structural feature of eukaryotic nucleus, the nucleoli, is prominently visible after *in situ* NuMat preparation (Figure 2d). The complete removal of chromatin (absence of DAPI staining) confirms the efficiency of extractions.

Previous studies on NuMat preparations on cell lines have shown that formaldehyde treatment is essential for TEM visualization of the characteristic fibrillar meshwork of NuMat. However, it is not known if formaldehyde crosslinking and stabilization is essential for *in situ* NuMat preparations in *Drosophila* embryos as well. In order to access the necessity of these treatments, we excluded them from the protocol. As seen in Figure 2, the overall shape and morphology of nuclei remains intact even if the fixation or stabilization steps are omitted from the NuMat preparation protocol. Complete removal of chromatin (no DAPI staining) indicates that NuMat is successfully prepared. However, internal lamin is not seen in uncrosslinked as well as unstabilized NuMat preparation. On the other hand, if the nuclei are over-fixed with formaldehyde, it is difficult to extract the digested chromatin efficiently. As seen in Figure 2, DAPI stained clumps of un-extracted chromatin remain stuck in the NuMat and internal lamin is also not sufficiently revealed. Thus, we observe that optimal crosslinking and stabilization steps are critical for a good NuMat preparation as based on Lamin Dm0 visualization, the *in situ* NuMat prepared after crosslinking and stabilization appears to be more intact. However, crosslinking and stabilization can be excluded altogether from the protocol, if the protein of interest is sufficiently retained in NuMat even without these treatments.

### In situ *NuMat preparation is extended to late embryos and larval tissue*

**Figure 3.**
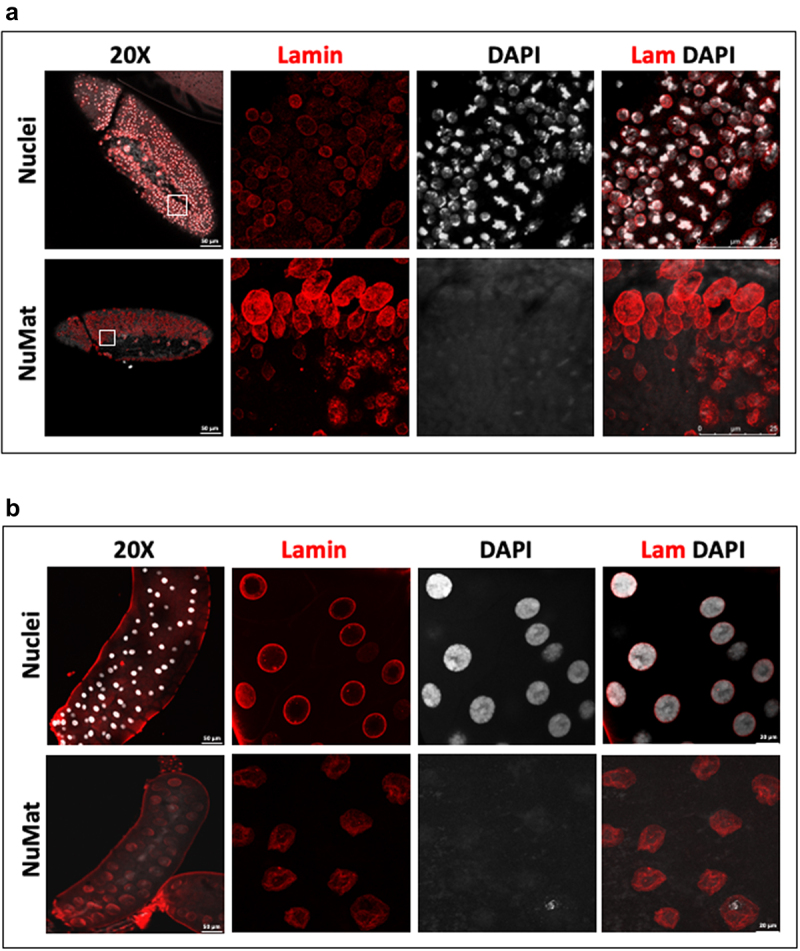
*In situ* NuMat preparation protocol works efficiently with late *D. melanogaster* embryos and larval tissues. **A**. *In situ* NuMat prepared with embryos at late stage of development shows that the digestion and extraction of chromatin (assessed by loss of DAPI staining) works well with different types and layers of cells present in a developing and differentiating embryo. Intra-nuclear Lamin Dm0 is revealed after the nuclear matrices are prepared. Boxes in 20X show the regions of embryo chosen for 63X imaging. **B**. *In situ* NuMat prepared with *D. melanogaster* third instar larval salivary glands shows that the bulk of chromatin present in polytene chromosomes is efficiently extracted (assessed by loss of DAPI staining) to reveal the nuclear architecture of salivary gland nucleus.

The strength of *in situ* protocol lies in the proposition that the power of *Drosophila* genetics can be harnessed to uncover novel molecular players with a role in nuclear architecture. However, in *Drosophila*, the effects of a genetic manipulation may not manifest in the nuclei of early embryos because of masking due to maternal deposition of the molecule of interest in the embryo. In such cases, the phenotype would be visible only at later stages of embryonic development. This necessitates that the protocol works effectively in older embryos or specific tissues in the larval or adult stages. As seen in Figure 3, the chromatin digestion and salt extraction work well in older embryos (Figure 3) as well as larval salivary glands (Figure 3). Negligible staining with DAPI indicates that bulk of chromatin is effectively removed and Lamin Dm0 in the nuclear interior is revealed defining the NuMat. Interestingly, the polytene nuclei lose its shape and give the impression of an ‘empty bag’ after removal of bulk of chromatin and NuMat preparation.

### *Use of* in situ *NuMat preparation to study dynamics of proteins during mitosis*

The early *Drosophila* embryo is a treasure-trove of interesting biological phenomenon. The nuclei in the syncytial embryo undergo 13 rounds of division. The cell cycle lasts only for 8 min during the early stages and progressively slows down to 18 min for the cycle 13 [13]. The rapid nuclear divisions at this stage increase the probability of catching an embryo with mitotic wave at the surface. It is possible to fix embryos during such a mitotic wave and study the cell cycle related dynamics of a nuclear constituent of interest.

**Figure 4.**
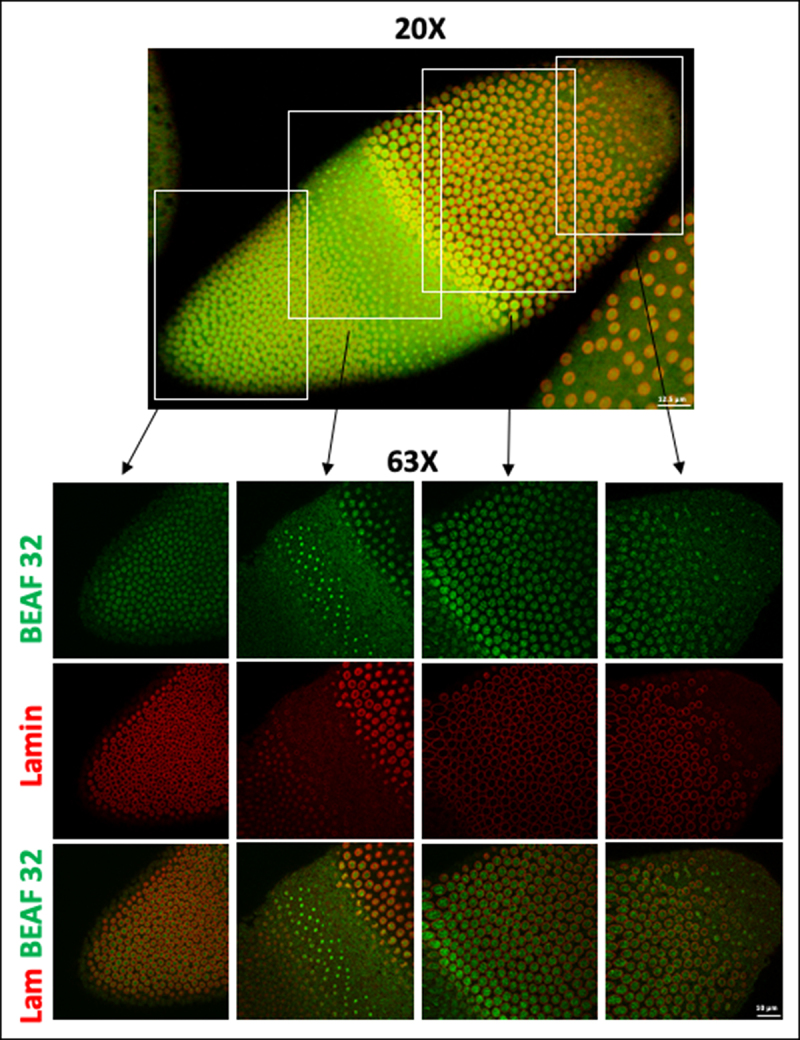
*In situ* NuMat prepared with early *D. melanogaster* embryo with a mitotic wave. *In situ* NuMat prepared with early syncytial embryos, captures a snapshot of an embryo with nuclei at different stages of mitosis. Immuno-staining with anti-Lamin Dm0 and anti-BEAF 32 reveals the dynamics of these nuclear proteins at different mitotic stages. A subset of BEAF 32 stays associated with mitotic nuclei even when the nuclear envelope (defined by Lamin Dm0) is dissolved.

Here we take an example of the protein BEAF 32 (Boundary Element Associated Factor), a known NuMat associated protein, to show how the association of this protein with nuclear architecture varies through cell cycle. BEAF 32 bound DNA boundary elements are known to tether to nuclear architecture by virtue of the interaction of the protein with NuMat [14]. *In situ* NuMat prepared on an early embryo that had a mitotic wave on display, shows that BEAF 32 remains associated with nuclear architecture at different stages of mitosis (Figure 4).

### *Use of* in situ *NuMat preparation in conjunction with fly genetics*

One of the common strategies used to study the possible role of a candidate protein in nuclear structure and function is to immuno-stain the protein for its localization. Disruption/depletion of a particular protein by mutation/RNAi is also used as an effective tool for analyzing gene function. *Drosophila* as a model organism is well suited for such studies. A vast repertoire of tagged-fly lines available publicly, is a valuable resource that makes it possible to study a novel protein, without worrying about the availability of its antibody. The BDGP gene disruption collection that disrupts ~40% of fly genes, provides a public resource that facilitates the application of *Drosophila* genetics to diverse biological problems [15,16]. On similar lines, the Transgenic RNAi Project (TRiP) has generated transgenic RNAi fly stocks that use Gal4/UAS system to induce RNAi silencing of specific genes. Embryos from such fly lines can be used for *in situ* NuMat preparation for architectural studies. Our protocol is particularly useful as it permits the use of small number of genetically screened embryos (typically ~50 in number) for the study.

**Figure 5.**
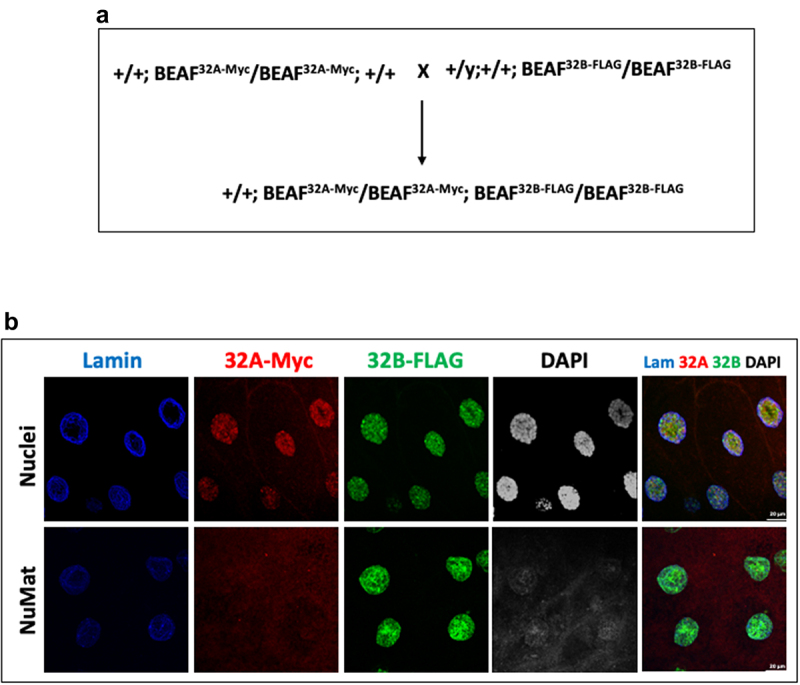
*In situ* NuMat preparation protocol can be used in conjunction with fly genetics. **A**. Fly cross scheme to generate a fly line carrying tagged isoforms of BEAF 32 in the same fly. **B**. Immuno-staining of unextracted/*in situ* NuMat prepared salivary glands with anti-Lamin Dm0, anti-Myc and anti-FLAG antibodies. Myc-tagged BEAF 32A and FLAG-tagged 32B, colocalize on several bands of the polytene chromosome in the salivary gland nuclei. After *in situ* NuMat preparation, 32A gets extracted out and 32B remains associated with NuMat.

Here again, we take an example of differential association of isoforms of BEAF 32 protein with the nuclear architecture. BEAF 32 exists as two isoforms, 32A and 32B, that form a hetero-trimer to bind to the chromatin. The only antibody available for BEAF 32 (from DSHB – Developmental Studies Hybridoma Bank at University of Iowa, USA) does not differentiate between the isoforms. We, in our lab, have generated two transgenic fly lines one of which had Myc tagged BEAF 32A transgene in it and the other had FLAG tagged BEAF 32B transgene present in it. Crossing them as shown in Figure 5, gives flies with Myc tagged BEAF 32A on chromosome 2 and FLAG tagged BEAF 32B on chromosome 3 simultaneously, in the F1 generation. Such a fly helped us to study both the isoforms of BEAF 32 in a single nucleus. Salivary glands from 3^rd^ instar larvae from this fly line were stained with anti-Myc and anti-FLAG antibodies simultaneously. As seen in Figure 5, BEAF 32A and 32B colocalize on most of the sites on the polytene chromosome in an intact nucleus. However, after *in situ* NuMat preparation, BEAF 32A staining is reduced to negligible, while BEAF 32B is retained predominantly in the NuMat. This experiment shows that the two isoforms of BEAF 32 interact differently with the underlying nuclear architecture and the extraction process is specific and not random.

## Discussion

The formidable task of organizing almost a meter of DNA into a ~10 µM diameter cell nucleus, that too in a functionally poised manner, is facilitated by NuMat. This biochemically defined framework unifies the nuclear structure and function and virtually every step of gene expression is draped on this framework.

NuMat essentially retains the spatial and topological configuration of the nucleus sans chromatin-associated fraction – hence the prep is very useful for visualization of architectural and topological elements. The solubilization of chromatin and moving it away from the underlying NuMat unearthed the existence of transcription factories, replication hubs, DNA repair scaffoldings and splicing sites [6,17–19]. These findings dramatically changed our perception of subnuclear architecture by introducing the idea of membrane-less nuclear bodies. With recent advances in microscopy techniques, we are discovering that these functional bodies are liquid like and dynamic. With so many functions to deal with, composition of this nuclear substructure is evidently quite complex. Composition of NuMat varies from cell to cell, far more than cytoplasmic or chromatin fraction [20].

The protocols used to prepare NuMat include steps to extract chromatin and soluble proteins. These methods of extraction are criticized because of their potential to cause aggregation of proteins. Over the years refined extraction methods, including use of low salt and extraction after encapsulation in agarose under physiologically relevant salt concentration have revealed a very similar fibro-granular meshwork that has been given many names like nuclear scaffold, nucleo-skeleton, nuclear cage and nuclear matrix. These experiments also provide wealth of data regarding the variations observed in the NuMat, when choice of salts/detergents or sequence of extraction is altered. However, all studies invariably agree on one point where a distinct fibro-granular structure is observed upon the removal of bulk of chromatin. Further studies have linked NuMat functionally to all of the nuclear processes. The DNA needs to interact with the sites where nuclear functions are performed (in the NuMat) and this necessity forms the basis of another layer of regulation of chromatin function. The regulation of chromatin function by nuclear architecture defines the concept of ‘spatial epigenetics’[21]. However, the specific molecular players still remain unexplored. The NuMat as a nuclear substructure remains an enigma due to the limitations posed by the lack of a screening technique, where the queried component is removed/overexpressed and its effect on nuclear architecture is visualized. Limitations are further enhanced by the technicalities involved in imaging NuMat. Live imaging has not been possible, as most of the NuMat components remain obscured by chromatin. On the other hand, looking at an extracted isolated nucleus or cells in culture also has limited utility.

Here we present a method for visualizing structural components of the nucleus, in the background of an experimental screening setup. Our method for *in situ* NuMat preparation makes it possible to study nuclear architecture in conjunction with the vast genetic resources of *Drosophila*. It allows the observation of NuMat in the *in vivo* context of a developing *Drosophila* embryo which has been very challenging until now. It can thus be used to study the cell cycle–related dynamics of the molecule of interest. The technique can also be used to study nuclear architecture in various other *Drosophila* tissues like the salivary glands, imaginal discs, etc. Once the *in situ* NuMat has been prepared, this technique is not only limited to immuno-staining and TEM but can also be used for DNA and RNA-FISH experiments. We have previously demonstrated such an application by studying the role of AAGAG RNA in nuclear architecture by using this technique [22].

In an earlier report, a method developed by Capco et al. [23] used whole cell mounts of 3T3 cells and extracted in buffers with physiological ionic strength without isolating nuclei. While this method has also been referred to as *in situ* NuMat, as it extracts the whole cell to derive the NuMat and no nuclei purification step is involved [24], our’s applies the classical biochemical techniques for making NuMat and extends it to intact *Drosophila* embryos and tissues. This adoption enables the visualization of nuclear architecture, in the background of an experimental setup, and compliments biochemistry, cell biology with *Drosophila* genetics.

Our protocol for *in situ* NuMat preparation is simple and does not require advanced technical knowledge. It can be performed by any researcher with basic experience in molecular biology. Further comparison with other existing protocols for NuMat preparation illustrates that the *in situ* NuMat preparation has several advantages. For instance, most protocols of NuMat preparation either involve isolation of pure nuclei from tissues or use cell lines, which have been grown in cell culture for a number of passages. Isolation of pure nuclei requires expertise, is time consuming and technically challenging, and often requires specialized equipment such as homogenizers and ultracentrifuges. On the other hand, cultured cells are often grown as 2D monolayers, which does not entirely reflect *in vivo* condition because of the lack of 3D cues [25,26]. However, our protocol does not require isolation of pure nuclei which reduces the time of preparation, hence minimizing the possibility of adventitious crosslinking by sulfhydryl oxidation that has been reported for lengthier protocols [27], and is easily adaptable. Moreover, our technique uses the whole developing embryo to prepare NuMat *in situ*, thereby causing minimal disturbance and thus reflects the *in vivo* conditions most faithfully. Taken together, we suggest that *in situ* NuMat preparation will facilitate in-depth analysis of nuclear architecture in the context of nuclear functions. An important advantage of our protocol is the requirement of low amount of sample, and therefore can be used to answer biologically relevant questions with very few embryos obtained by a genetic screen, in contrast to the large amount of tissue, embryos or cells required for biochemical experiments [28–31]. As this technique uses whole embryos without the isolation of pure nuclei, our protocol may not be suitable for biochemical experiments such as westerns, proteomics, etc.

Overall, the advantages of our methodology as compared to previously published protocols can be summarized as (1) a quick protocol which does not require nuclear isolation and thus reduces the chances of artefactual alterations in the nucleus, (2) can be used on whole organism thus facilitates contextual observations *in situ*, (3) can be used on dissected tissue thus can be applied to study *in situ* tissue-specific nuclear architecture, (4) requires small quantities of starting material thus permits the use of genetically altered samples which are tedious to procure, (5) facilitates visualization of components of various nuclear functions like replication, transcription, DNA repair and splicing, in the nuclear architectural context in the embryo. These components otherwise remain shielded by chromatin and are difficult to study. The protocol is a powerful means to define components of nuclear architecture and analyze their function.

## Materials and methods

The main experimental steps of *in situ* NuMat preparation have been outlined in the form of a workflow in Figure 1. A detailed step by step protocol has been submitted as Supplementary document 1.

### Drosophila *embryo/tissue collection, fixation and permeabilization*

*Drosophila* embryos (0–2 hr-old – 50–100 embryos) or of desired developmental age were collected on a grape juice-agar collection plate. The collected embryos were dechorionated with 50% sodium hypochlorite and washed thoroughly with running tap water. The dechorionated embryos were then devitellinized and fixed simultaneously in a mix of 4% formaldehyde in phosphate buffered saline (PBS) and heptane (in 1:1 v/v ratio). The embryos were vigorously shaken in the fixative:heptane mix for 20 min at room temperature (RT). The aqueous layer at the bottom was removed with a pipette and the embryos were further devitellinized using a mixture of 1:1 (v/v) ice-cold methanol:heptane. The tube was shaken vigorously until the embryos start to settle at the bottom of the tubes. Embryos that are devitellinized completely sink to the bottom, while the damaged ones and the ones that have vitelline membrane still attached, remain floating. This step was repeated several times. The devitellinized embryos were then equilibrated in aqueous media with several washes in PBS + 0.1% Triton-X-100 (PBT). Some embryos were reserved to serve as unextracted controls while the rest were used for NuMat preparation.

Efficient dechorionation and devitellinisation of embryos is a critical step of the protocol. Improper removal of chorion makes the embryo impermeable to most of the treatments, thus compromising NuMat preparation. Fixation is another important step for *in situ* NuMat preparation. Over-fixation may lead to artefactual attachment of molecules to NuMat, whereas under-fixation may cause extraction of genuine components. Here, we use 4% formaldehyde for fixation but alternative fixatives such as paraformaldehyde may be used, in which case the time required for fixation must be empirically determined. We have also isolated NuMat without fixation and observe that the nuclear shape, size and architecture, as revealed by Lamin Dm0 immuno-staining, remains intact. Thus, the decision to fix or not to fix would depend on how labile the queried component is. However, coagulating fixatives such as ethanol or long-range fixatives such as DSG (Disuccinimidyl glutarate), EGS {Ethylene glycol bis(succinimidyl succinate)}, etc. should be avoided because they may cause artifacts.

For *in situ* NuMat preparation from salivary glands, the desired tissue was dissected out from 3^rd^ instar larvae and washed in PBS (20 pairs of glands). The tissue was then fixed with 4% formaldehyde in PBT for 20 mins at RT. Fixative was removed by washing thrice with PBT. For each wash PBT was added to the tube and the tissue was allowed to settle with gravity following which the PBT was removed. Alternatively, the washings and other manipulations of the dissected tissue was done in a chambered slide under the view of an inverted microscope, to avoid the loss of tissue during processing.

### In situ *NuMat preparation*

To prepare NuMat, fixed embryos/tissue were stabilized by incubating for 20 mins at 37°C in PBT. This step stabilizes the nuclear architecture and facilitates the isolation of NuMat with comparatively intact composition as compared to unstabilized embryos/tissue. After stabilization, the embryos/tissue were extracted sequentially with salt and nonionic detergent. Extraction was carried out first in 0.4 M NaCl followed by 2 M NaCl along with 0.5% Triton-X-100. During this step, most of the nucleoplasmic proteins are extracted. The high salt treatment removes majority of histones leading to unpackaging of DNA. The loosened DNA protrudes out from the nuclear margin and appears as a halo when stained with DNA dyes like DAPI. Salt extraction was followed by washes in PBT and then the DNA was removed by extensive digestion with DNase I. This treatment removes most of the DNA and chromatin-associated components. The embryos/tissue were finally washed with PBT. These embryos/tissue containing *in situ* NuMat were further processed for immuno-staining, immuno-FISH or transmission electron microscopy (TEM) using standard protocols to visualize the queried component of nuclear architecture.

## Supplementary Material

Supplemental Material

## Data Availability

The authors confirm that the data supporting the findings of this study are available within the article and its supplementary materials. Any additional data required are available from the corresponding author (RKM), upon reasonable request.
