# Supplementary material for: In situ nuclear matrix preparation in Drosophila melanogaster embryos/tissues and its use in studying the components of nuclear architecture
Source: Nucleus. 2022 Mar 3;13(1):116–28. doi: 10.1080/19491034.2022.2043608 (PMC8896195; doi:10.1080/19491034.2022.2043608)
Supplement: Supplemental Material [file KNCL_A_2043608_SM7393.docx]

**Detailed Materials and Method**

**Materials**

1. **Reagents**

- DAPI (Sigma-Aldrich cat. no. D8417)
- Deionized water
- DGD (Electron Microscopy Sciences cat. no. 13255)
- DNase I (Sigma-Aldrich cat. no. D4527)
- EDTA (Sigma-Aldrich cat. no. E9884)
- Ethanol-EM grade (Electron Microscopy Sciences cat. no. 15055)
- Formaldehyde (Sigma-Aldrich cat. no. F8775)
- Glutaraldehyde-EM grade (Sigma-Aldrich cat. no. G5882)
- Hepes (Sigma-Aldrich cat. no. H3375)
- Heptane
- Isopropanol
- KCl (Sigma-Aldrich cat. no. P9541)
- KH_2_PO_4_ (Sigma-Aldrich cat. no. P5655)
- Methanol
- Na_2_HPO_4_ (Sigma-Aldrich cat. no. S3264)
- NaCl (Sigma-Aldrich cat. no. S3041)
- N-butyl alcohol (Electron Microscopy Sciences cat. no. 11920)
- OsO_4_ (Electron Microscopy Sciences cat. no. 19192)
- Parafilm
- Pipes (Sigma-Aldrich cat. no. P1851)
- PMSF (Sigma-Aldrich cat. no. P7626)
- Primary antibody against the protein to be probed
- Secondary antibody conjugated with fluorescent probe
- Sodium cacodylate (Electron Microscopy sciences cat. no. 11654)
- Sodium Hypochlorite solution (Fisher Scientific product. No. 27908)
- Sucrose (Sigma-Aldrich cat. no. S9378)
- Trizma (Sigma-Aldrich cat. no. T6066)
- Triton X-100 (Sigma-Aldrich cat. no. T8787)

1. **Equipment**

- Chemical fume hood
- Confocal microscope
- Coverslips
- Fine forceps
- Formvar coated copper grids
- Glass knives
- Glass slides
- Heating block
- 37°C incubator
- 60°C incubator with rotation capability
- Loop and Handle
- 1.5ml, 0.5ml and 0.25ml microfuge tubes
- Nail polish (clear)
- NaOH
- Needle
- Paint brush
- Petri plates for embryo collection
- Pipette
- pH meter
- Rotator
- TEM (Transmission Electron Microscope)
- Ultramicrotome
- Vials for maintenance of *Drosophila* lines

1. **Solution preparation**

**0.5 M EDTA**

- Add 14.6g EDTA to 60ml deionized water. While stirring slowly adjust pH to 8 with 10N NaOH. EDTA will dissolve as the pH of the buffer comes close to 8. Filter and store at room temperature.

**1M HEPES**

- Add 23.8g HEPES to 80ml of deionized water. Dissolve and adjust pH to 7.5 with 10N NaOH. Adjust volume to 100ml. Filter and store at room temperature.

**0.1 M PMSF**

- Dissolve 174.1mg PMSF in 8ml isopropanol. Adjust volume to 10ml with isopropanol. Filter and store at 4° C.

**10% Triton X-100 (v/v)**

- Add 5ml 100% triton X-100 to 45 ml deionized water. Gently mix and store at room temperature.

**10X PBS**

- Dissolve 80g of NaCl, 2g of KCl, 14.2g of Na_2_HPO_4_ and 2.4g of KH_2_PO_4_ to 800ml deionized water. Adjust pH to 7.4 using 0.1N HCl. Adjust volume to 1L. Dilute 10X to 1X with deionized water for use.

**Fixation buffer**

- 1X PBS containing 4% formaldehyde (v/v)

**PBT**

- Add 1ml of 100% Triton X-100 to 199ml of 1X PBS and gently mix until all the Triton X-100 has dissolved. The final concentration of Triton is 0.5% (v/v).

**Extraction Buffer I (EBI) (containing 0.4M NaCl)**

- Add 5µl of 1M HEPES (pH - 7.4), 0.6µl of 3M KCl, 4µl of 0.5M EDTA (pH – 8.0), 80µl of 5M NaCl, and 10µl of 100mM PMSF to 900µl of deionized water.

**Extraction Buffer II (EBII) (containing 2M NaCl)**

- Add 5µl of 1M HEPES (pH - 7.4), 0.6µl of 3M KCl, 4µl of 0.5M EDTA (pH – 8.0), 400µl of 5M NaCl, and 10µl of 100mM PMSF to 580µl of deionized water.

**Digestion Buffer (DB)**

- Add 10µl of 1M Tris, 6.6µl of 3M KCl, 14µl of 5M NaCl, 10µl of 1M MgCl_2_, 50µl of 10% Triton X-100, 1µl of 0.1M PMSF

**Method**

**Steps (1-5) *Drosophila* embryo collection and dechorionation**

1. Set up embryo collection cages with 50 male and 100 female flies (2-10 days old) and incubate at 25°C overnight to acclimatize the flies to the new environment. This step is important to maximize the number of eggs laid. In fly lines with low oviposition frequency, multiple cages can be set up parallelly.
2. Replace the old embryo collection plate with a fresh one and incubate at 25°C for 1hr. Discard the embryos laid in embryo collection plates in step 1 and 2.

- **CRITICAL** Step 2 is important to ensure that the embryos are synchronized and of a particular developmental stage

1. Replace the previous embryo collection plate with a fresh one and incubate at 25°C for 2 hrs.
2. Collect embryos (50-100 embryos is a good number) by pouring tap water on the embryo collection plate and collecting it into a beaker with a brush.
3. Dechorionate embryos by treating with 50% bleach for 2-3 minutes and wash thoroughly with tap water straining through a mesh cloth till the smell of bleach is no longer detectable.
   - **CAUTION** Sodium hypochloride is toxic and can de-stain clothes. While handling wear separate gloves and lab coat.
   - **CRITICAL** Dechorionation is critical for the rest of the protocol since chorion of the embryo forms a barrier and will inhibit all access to reagents in later steps. It can be assessed by observing embryos under the microscope. It is very important to remove residual bleach by rinsing thoroughly with tap water since bleach is a strong oxidizing agent and may interfere with reactions in the following steps.

**Steps (6-10) Fixation and Devitellinization of embryos**

1. Rinse embryos twice with 1X PBS. Collect embryos in a 1.5ml Eppendorf tube and keep on ice for 10 mins to chill. Size of tube and volume of fixative should be decided according to the number of embryos processed. For about 50-100 embryos, a 1.5ml Eppendorf tube can be used. For higher numbers (>500 to 0.5gm), a 15ml screw cap polypropylene tube can be used. The tube should be filled 80% with fixative/heptane mix, to avoid loss of embryos due to sticking on the walls. The protocol detailed below is for ~100 embryos that can be processed in a 1.5ml Eppendorf tube.
2. To fix, submerge embryos in 0.6ml of fixation buffer (1X PBS containing 4% formaldehyde) and add equal volume of heptane (0.6ml). Mix by vigorous shaking and then incubate the tube on a rotating wheel at 100rpm at room temperature for 20 mins. The NuMat isolation can be done without fixation and for this formaldehyde should not be added to the devitellinization mix.
   - **CAUTION** Formaldehyde is toxic. Always use in a fume hood.
3. Let the tube stand on ice for 2 mins to allow separation of the aqueous and organic phases. Gently aspirate and discard the lower aqueous phase without sucking any embryos in the process.
   - **CRITICAL STEP** The fixed embryos form a ring at the junction of aqueous solution and heptane.
4. To devitellinized the embryos, add methanol (pre-chilled at -20°C, volume equal to heptane). Shake vigorously and let devitellinized embryos settle at the bottom. Gently aspirate and discard supernatant.
   - **CRITICAL STEP** It is very important to add pre-chilled methanol and to shake the tube vigorously after adding methanol for proper devitillinization of the embryos. After devitillinization, the embryos should appear whitish in colour as compared to non-devitellinized embryos which appear yellowish in colour. Devitellinized embryo sink to the bottom of the tube, and embryos with vitelline membrane still attached, float at the surface or interface.
5. Wash these embryos twice, in quick succession, by adding 1 ml pre-chilled methanol, allowing the embryos to settle down and then discarding the supernatant by gentle aspiration.

- **CRITICAL STEP** In all the steps of washing embryos, give time for embryos to settle down completely and then pipette out the solution completely from the tubes for preventing the loss of sample in the procedure. Fine tip should be used to aspirate solutions since it prevents loss of embryos.
- **PAUSE POINT** These dechorionated, devitellinized and fixed embryos can be safely stored at -20°C in methanol and used for one month. This step is useful for pooling embryos of lines with low rate of oviposition.

**Steps (1a-5a) Dissection and fixation of larval tissues**

1a. Transfer the 3^rd^ instar larvae of desired phenotype with the help of a brush in a watch glass with 1X PBS. Wash the larvae of any adhering food material.

2a. Dissect out the desired tissue (salivary glands or imaginal discs) with the help of dissecting needles and forceps. Dissecting 20 larvae to yield 20 pairs of salivary glands/20 pairs of different imaginal discs is good enough for the purpose.

3a. It is good to keep the glands/discs in bunches without separating them in individual entities, as this prevents their loss. They can be separated after the completion of the whole process, just before mounting on a slide for observation. Also place the dissected tissues in chambered slides. Usually, it is difficult to view dissected tissues in an Eppendorf tube and this results in loss of material during pipetting steps. Thus fixation, washings and all the processes from step 11 to 19 for dissected tissues can be carried out in chambered slide, under the view of an inverted microscope. In such a situation, instead of using a rotating wheel, a platform shaker should be used for shaking during incubation. Addition or removal of solution should be done while keeping the tip of pipette tip in view and avoid sucking out of the tissue. This prevents the loss of any material during processing.

4a. Remove the PBS and replace it with fixation buffer (1X PBS containing 4% formaldehyde) and incubate it at room temperature for 20 mins.

5a. Remove the fixation buffer and proceed to step 11 as detailed below.

**Steps (11-19) *In situ* NuMat preparation**

1. Allow embryos to settle down and discard methanol by gentle aspiration. Wash the embryos by filling up the tub with 1ml PBT and allowing the embryos to settle to the bottom, three times in quick succession. Fixed tissue is also washed with PBT three times.
2. Rehydrate the embryos three times for 20 minutes each with 1ml prechilled PBT with rotation at 40 rpm at room temperature. Such rehydration is not needed for tissues as they are already in aqueous medium.

- **PAUSE POINT** Aliquot few embryos/tissues in 0.5 ml of microfuge tube in 0.5 ml PBT and store at 4°C. Store two such aliquots if both immunofluorescence and TEM analysis are required. These aliquots serve as the controls which are not treated with salt extractions and DNase digestion. These untreated control embryos should be treated for immunofluorescence and TEM simultaneously with *in situ* NuMat from steps 20 and 28 respectively.

1. Add 1ml PBT to the embryos/tissues in 1.5ml microfuge tube and incubate the tube at 37^o^C for 20 minutes (Stabilization). This step can be omitted if stabilization not deemed necessary. Discard supernatant by gentle aspiration.
2. Add 1 ml EB I to the tube/chambered slide and incubate on rotating wheel/platform shaker at 40 rpm for 20 minutes at room temperature (salt extraction I).
3. Allow embryos/tissues to settle down and discard supernatant by gentle aspiration. Add fresh 1ml EB II and incubate at 40 rpm for 20 minutes (salt extraction II).
4. Wash embryos/tissues three times (10 minutes each) with PBT by adding the buffer and rotating/shaking the tube/chambered slide at 40rpm. Allow the tube to stand for 2 minutes to let the embryos settle at the bottom and remove the buffer by careful aspiration after the embryos/tissues settle down to the bottom of the tube.
5. Allow embryos/tissues to settle down and discard supernatant. Add 100 µl of DB containing 2µl of DNase I (10mg/ml) and incubate at 37°C shaker incubator for 50 minutes at 200rpm.

- **CRITICAL STEP** the temperature should be maintained precisely at 37°C for efficient DNA digestion.

1. Repeat step 16 for washing. The settled embryos/tissues with *in situ* NuMat prepared are ready for observation.
2. Split embryos/tissues from 1.5 ml microfuge tube to two 0.5ml microfuge tube and proceed for immunofluorescence or TEM observations.

**Steps (20-26) Immunostaining of *in situ* NuMat**

1. Dilute primary antibody according to manufacturer’s instructions and add to untreated control embryos/tissues and *in situ* NuMat in 0.5ml PBT, seal tubes with parafilm and incubate with rotation for 3 hours at room temperature.

- **PAUSE POINT** Alternatively, the primary antibody incubation can also be carried out overnight (12-16 hrs) at 4°C with rotation.

1. Repeat step 16 for washing.
2. Dilute secondary antibody according to manufacturer’s instructions and add to untreated control embryos/tissues and *in situ* NuMat in 0.5 ml PBT, seal tubes with parafilm and incubate with rotation for 3 hours at room temperature.

- **CRITICAL STEP** secondary antibody is fluorescently labelled so all the steps following this till step 26 should be done in such a way that the sample is not exposed to direct light. For this, the tubes in which the secondary incubation is done should be well wrapped with aluminium foil to prevent any contact with light.

1. Repeat step 16.
2. After the last wash with PBT, allow the embryos/tissues to settle and discard supernatant with gentle aspiration. Transfer embryos/tissues on a glass slide by adding a small amount of PBT and pipetting the embryos/tissues along with the solution.

- **CRITICAL STEP** Use cut tips for pipetting out these embryos from the microfuge tubes and putting them on glass slides. Once the embryos have been transferred on the slide, discard excess solution from the slides with gentle aspiration without losing the embryos in the process.

1. Add mounting media along with DAPI in the required quantity.
   - **CAUTION** DAPI is potentially carcinogenic. Handle with care.
2. Gently place the coverslips with the help of a needle on top of the embryos/tissues preventing any air bubble to enter. Care should be taken not to squash the embryos/tissues. Seal the coverslip from sides by gently applying a transparent nail polish on all four sides of the cover slip.

- **CRITICAL STEP** These slides should be stored in boxes or slide book where they are not in contact with direct light.
- **PAUSE POINT** The slides can be scanned immediately or stored overnight at 4°C in slide boxes or slide books. However, these should be scanned within one day of preparation.

1. The slides can be scanned using a confocal or multiphoton microscope. Here we have used Leica SP8 microscope to image the slides. All the images were taken with 20X and 63X objectives. The acquired images were processed using LAS X software to generate the projection images.

**Steps (28-48) Preparing sample for TEM**

1. Fix untreated control embryos and NuMat carrying embryos from steps 12 and 19 respectively by adding 2% glutaraldehyde in 0.1M cacodylate buffer and incubating samples with rotation for 1 hr at 4°C.
   - **CAUTION** Glutaraldehyde is toxic. Handle with care in a fume hood.
2. Let embryos settle down and discard supernatant by gentle aspiration.
3. Wash embryos with 0.1 M cacodylate buffer three times by adding the cacodylate buffer, incubating with rotation for 5 minutes at 4°C, letting the embryos settle down and discarding supernatant.
   - **PAUSE POINT** The samples can be stored at 4°C for 12-16hr in 0.1 M cacodylate buffer after glutaraldehyde fixation.
4. Postfix samples with 1% OsO_4_ in 0.1 M cacodylate buffer and incubate with rotation for 30 minutes at 4°C.
   - **CAUTION** OsO_4_ is extremely toxic. Handle with care in a fume hood.
5. Repeat steps 29 and 30.
6. Dehydrate samples with 35% ethanol in 0.1M cacodylate buffer and incubate with rotation for 10 minutes at 4°C.
7. Allow samples to settle and discard supernatant.
8. Repeat steps 33 and 34 with following concentrations of ethanol: 50%, 70%, 80%, 90%, 95%, and 100%. Repeat the wash in 100% for one more time.
9. Add ethanol:n-butyl alcohol (nBA) in 2:1 ratio and incubate with rotation at room temperature for 10 minutes.
10. Allow samples to settle and discard supernatant by gentle aspiration. Add ethanol:nBA in 1:2 ratio and incubate with rotation at room temperature for 10 minutes.
11. Allow samples to settle down and discard supernatant by gentle aspiration. Add 100% n-BA and incubate with rotation at room temperature for 10 minutes.
12. Allow samples to settle down and discard supernatant. Add n-BA:DGD in the ratio 2:1 and incubate with rotation at 60°C for 1hr.
13. Allow samples to settle down and discard supernatant. Add n-BA:DGD in the ration 1:2 and incubate with rotation at 60°C for 1hr.
14. Allow samples to settle down and discard supernatant. Add 100% DGD and incubate with rotation for 1hr.
15. Repeat step 41.
16. Incubate samples along with the DGD at room temperature to allow it to solidify. If samples are stuck on the side of the tube bring them to the bottom by pipetting little amount of DGD on the sides of the tube.

- **PAUSE POINT** The DGD embedded samples can be stored indefinitely at room temperature.

1. Cut the microfuge tubes with a sharp blade and take out the DGD block containing sample.
2. Trim the sample and cut 70-90 nm thick sections using glass knife on an ultramicrotome at an angle of 10° and float the sections on water.
3. Transfer the sections on a formvar coated copper grids using a loop and handle and allow to dry for 2 hours.
4. Remove embedding DGD by dipping the copper grids containing sections in n-BA and incubating for 12-16hrs.
5. Dry grids. Observe and capture images using TEM at 120V.
   - **CRITICAL** Steps 44-48 should be done in dust free environment to avoid contamination of sections and EM grids.

- **TIMING**

Step 1-10, dechorionation, devitellization and fixation of the embryos : ~ 1 hour.

Step 1a-5a, dissection of larval tissue : ~2 hour

Step 11-19, processing of the embryos for NuMat preparation : ~ 4.5 hours.

Step 20-26, antibody staining and mounting of embryos on slides : ~ 9 hours-1 day.

Step 27, scanning of the slides : ~ 2 hours - 1 day.

Steps 28-43, embedding samples in DGD for sectioning : ~9 hours

Steps 44-46, sectioning of samples using ultramicrotome : ~ 5 hours

Step 47: removal of DGD : ~12-16 hours

Step 48: imaging with TEM : ~4 hours
